# Supplementary material for: Complexome profiling of the Chlamydomonas psb28 mutant reveals TEF5 as an early PSII assembly factor
Source: Plant Cell. 2025 Mar 17;37(6):koaf055. doi: 10.1093/plcell/koaf055 (PMC12164590; doi:10.1093/plcell/koaf055)
Supplement: koaf055_Supplementary_Data [file koaf055_supplementary_data.zip › psb28_interactive.zip!/plots/intensity/qualityPlots/peptides/DXS1_Cre07.g356350.t1.1.html]

### Cre07.g356350.t1.1

Trivial name: DXS1  
SeqCoverage [median]: 29.90  
Mass: 78.82 kDa  
  
 

| Sequence | MissedCleavage | Mass [Da] | wtIntensity | muIntensity | Score |
| --- | --- | --- | --- | --- | --- |
| ATAKIDEYAR | 1 | 1136.58 | 0.00 | 2547.36 | 72.65 |
| DGVSTTVIDAR | 0 | 1132.57 | 9685.02 | 17326.67 | 142.43 |
| DLKGVPLEVGK | 1 | 1153.67 | 1470.78 | 3174.60 | 99.50 |
| DLKGVPLEVGKGVVRR | 3 | 1721.03 | 0.00 | 1375.18 | 50.61 |
| DQLAMAGLTSQHIASTALTTLGR | 0 | 2355.22 | 0.00 | 1940.56 | 61.35 |
| FRPMTLPDR | 1 | 1131.59 | 3865.97 | 12057.08 | 101.65 |
| FSLSALQA | 0 | 835.44 | 1332.63 | 0.00 | 94.01 |
| GVPLEVGK | 0 | 797.46 | 5255.78 | 8597.96 | 114.72 |
| GYDQIVHDVSLQK | 0 | 1500.76 | 3912.55 | 17397.98 | 101.39 |
| GYDQIVHDVSLQKLPVR | 1 | 1966.06 | 0.00 | 850.52 | 41.87 |
| GYLPAETAQDK | 0 | 1191.58 | 0.00 | 3571.96 | 85.73 |
| GYLPAETAQDKMHGVVK | 1 | 1842.93 | 4287.71 | 14474.89 | 92.94 |
| IDEYAR | 0 | 765.37 | 5557.99 | 8978.33 | 131.65 |
| IVAVHAAMAGGTGLYR | 0 | 1585.84 | 6837.63 | 12674.98 | 114.50 |
| IVAVHAAMAGGTGLYRFEK | 1 | 1990.05 | 0.00 | 2472.51 | 49.22 |
| NFNNEQLK | 0 | 1005.49 | 280.62 | 1947.16 | 89.55 |
| NMIVILNDNQQVSLPTQYNNK | 0 | 2445.23 | 0.00 | 950.32 | 66.78 |
| NQDPVGALSSALAR | 0 | 1397.73 | 7780.03 | 27474.53 | 234.58 |
| QLPDVVQK | 0 | 925.52 | 0.00 | 5302.60 | 105.04 |
| SAETVGPVLVHVVTEK | 0 | 1663.91 | 8978.44 | 56399.66 | 120.87 |
| SDIVHTVSR | 0 | 1012.53 | 0.00 | 2532.16 | 56.73 |
| TPLLDTVNYPVHLK | 0 | 1608.89 | 2743.61 | 49437.55 | 180.48 |
|  |  |  | 61988.8 | 251484.5 |  |
